# Assessment of hospitals' websites in Portugal

**DOI:** 10.3389/fpubh.2022.995153

**Published:** 2022-08-26

**Authors:** Demetrios Sarantis, Delfina Sa Soares, Joana Carvalho

**Affiliations:** The UNU Operating Unit on Policy-Driven Electronic Governance (UNU-EGOV), Guimaraes, Portugal

**Keywords:** health, e-health, assessment, online communication evaluation, wellbeing, digitization of user experience, social welfare

## Abstract

Technological advances have highly facilitated the accession of health-related information. As the public search on hospital websites for information and services is increasing, hospitals need to upgrade their websites to meet the high standards and demands of health-care consumers. Today, many hospital websites use a patient-centric approach to promote engagement and encourage interaction for better health-related decision making. However, little is known about the current state of hospital websites in Portugal. This study aims to assess hospital websites in Portugal and offer improvement insights. In this paper, the websites of 132 selected hospitals were thoroughly examined and assessed according to a predefined list of indicators and sub indicators, based on four criteria: technology, content, services and community interaction, defined in the Health Sector Website Assessment Index (HSWAI) instrument. Most of the websites scored satisfactorily in the technology criterion, performed fairly well in providing fundamental content, but showed shortcomings in quality metrics data and online patient services, and most of them fell short in community interaction elements. Overall, the results show that each hospital website must be improved in specific features in order to become effective and efficient. Several of the identified assessment elements (indicators/sub indicators) fall under Sustainable Development Goal (SDG) 3, United Nations health-focused goal, and could be used by governments to assess their progress toward achieving that specific goal. Therefore, this study not only provides a comprehensive and systematic approach that quantitatively measures hospital websites' overall performance, but also contributes to practical applications in terms of worthwhile recommendations for a website that meets patient's demands and hospital's operational needs.

## Introduction

To ensure healthy lives and promote well-being for all at all ages is the motto of Sustainable Development Goal 3 (SDG 3). Achieving such a goal demands broad coverage of health services and health related information, which must be provided by credible health institutions as hospitals. Communication dissemination of hospitals must be done consistently making use of their online presence, counting on professional websites that convey useful, timely, and correct information but also, that offer personalized services to their patients. There has been a growing effort from health institutions to design strategies for the implementation of more robust electronic services (1) to increase patient satisfaction and strengthen trust in health sector institutions (2).

The significant spread of internet usage has led to more patients opting to use healthcare websites for information about their illness or treatment, participate in support groups, searching and consulting with the health community (3, 4). This increase is bred by the rapid development of telecommunication technologies and affordable access to internet (5, 6), thus providing opportunity for hospitals to engage their patients via informative and educational web-based systems.

Health services' users scrutinize health care costs by comparing different health services provided by different agencies and save time and money by streamlining communications with different healthcare providers. Healthcare institutes' websites can facilitate better patient-centered care via improving healthcare procedures, clinical results provision, patients' requirements and preferences satisfaction, collaborative decision-making, interaction between doctors and patients, and access to medical data (7–9). This raises efficiency, reduces medical errors, improves disease management and decreases healthcare system load, reduces overcrowding in hospital and inappropriate health interventions. It also creates a new revenue opportunity, for health-care providers, which is an important financial aspect considering the limited monetary resources of the health sector (10, 11).

Therefore, healthcare website assessment has captured the attention of researchers, health organizations, decision makers and others responsible for designing, implementing, maintaining and studying the use and impact of web offered health services. Yet, little is known about the levels of Portuguese hospitals provided services through their websites. Therefore, it becomes meaningful to explore the current status of Portuguese hospital websites and assess them to ameliorate access to health related information as well as to broaden patient engagement. This study aims to systematically assess Portuguese hospital websites, by applying HSWAI, and provide suggestions to improve online services, patient engagement and access to health information.

Portugal has around 10 million inhabitants (12) and 238 hospitals (12) to serve its population according to data from the Portuguese National Statistics Institute regarding the year of 2019. Population has access to the public health system, but private hospitals also have high demand due to its faster service and agreements with many insurance companies and the public institute for protection and assistance in sickness (ADSE).

This paper is organized as follows. Section Related work presents assessment efforts conducted at national level. A concise illustration of the applied assessment instrument (HSWAI) and a brief contextualization of the Portuguese health sector follows in section Health sector website assessment index (HSWAI). Section Findings presents the findings of Portuguese hospitals websites' assessment. Finally, results are discussed in section Discussion and the paper recapitulates in conclusion.

## Related work

Assessment of healthcare websites has been investigated in several studies, at national level, from different perspectives and criteria. A systematic review of recent, after 2016, healthcare agencies' website assessment, has been conducted during this research. For the purposes of this paper, a set of publications, dealing with hospital website assessment at national level, have been researched, and reviewed.

Huerta et al. (13) assessed children's US hospitals Web and social media presence. The work concludes that by improving website design, complying with internet industry standards, and optimizing search engine performance, hospitals can maximize the potential power of the Internet to engage and inform patients.

Kaur et al. (14) found, during their investigation, that the Indian hospital websites are not mature enough and websites are neglecting performance and quality criteria and need lots of improvement in order to meet the expectation of people and motivate them to use the digital media for health-related information. Results showed that many Indian hospital websites have accessibility problems. Full compliance with accessibility WCAG 2.0 guidelines remained low. The majority of websites lack multilanguage features and the content of the website is difficult to read and understand. The security is also a concern due to the use of vulnerable content management systems. Indian hospitals require many improvements in their websites' design to increase the usability and security.

El Rahman's (15) and Alhadreti (16) studies' findings shows that unless barriers to accessibility and visibility are removed then usability of the Saudi Arabia hospitals' websites would be compromised. The results show that most hospitals pay minimal attention to the content accessibility and to the structure of provided information. They attribute these results to a lack of established legislation and guidance related to web accessibility in Saudi Arabia. The return on investment for the health websites would be maximally exploited if those prerequisites are given due recognition.

Alhuwail et al. (7), provide a comprehensive assessment of nine Kuwaiti in-patient hospitals using automated and expert-based tools and evaluation methods. Most of the websites fell short in all four dimensions, accessibility, usability, presence, and content. The websites focus primarily on promoting services provided by the hospital rather than engaging and communicating with patients or providing evidence-based information.

Bach et al. (17), examined the content of hospital websites in Bosnia-and-Herzegovina, Croatia and Slovenia. They found out that hospital websites are mostly used as information repositories and less as online communication channels. Additionally, they conclude that the country, which had standardized recommendations issued by the public authority, had more developed hospital websites.

Król and Zdonek (18) assessed the quality of 91 infectious disease hospital websites in Poland in light of the COVID-19 pandemic. The research has shown that most of the websites were of satisfactory quality, apart from those that were not mobile-ready. The research suggests that the quality of infectious disease hospital websites in Poland is significantly diversified in search engine optimization, mobile-friendliness, and needs of people at risk of digital exclusion.

Hong and Kim (19) scrutinizes the websites of four hospitals in Korea, Switzerland, Germany and in the United Arab Emirates. They concluded that each website uses a different way providing medical information, services and education. The differences include aspects such as decision-making methods, planning processes and e-health tool types. Each website applies different interactive tools such as traditional functional tools, core e-business tools, patient support tools, visitor related tools and emerging functional tools. They suggest that by targeting not only the patients but also the general website users will eventually improve health information accessibility.

Saghaeiannejad-Isfahani et al. (20) suggested that that none of the Iranian hospital websites are in desirable condition. This happens due to lack of consultation with experts, lack of attention to website design principles, and lack of content management and monitoring.

## Health sector website assessment index (HSWAI)

This section describes briefly the instrument applied and the application process in Portuguese context followed in the study.

### The instrument

Health Sector Website Assessment Index (HSWAI) is an instrument that assesses the hospital website according to four main criteria: Content, Services, Community Interaction, and Technology Features. HSWAI is a multi-criteria index composed of a series of indicators, the objective of which is the measurement of various aspects of health sector institutes' web presence. A detailed description of HSWAI can be found in Sarantis et al. (21). X. Thereinafter the four criteria are briefly presented.

Content criterion investigates fundamental elements for hospitals, which want to optimize services, facilitate patients, improve internal operation and maximize their profit. The criterion includes aspects such as what information needs to be provided, how information should be stored and organized, how information can be retrieved and how information should be displayed. The criterion considers health related content as a way to keep patients engaged with hospital's website and help them learn how they would benefit from its services.

Efficient electronic health services have the potential to improve service level, save time, reduce administration costs, increase communication efficiency, integrate patients in their health management and general contribute in the improvement of healthcare networks. Services criterion considers adoption and diffusion of digital services in hospitals' websites. Virtual laboratories, electronic patient folders, online medical services, user-centered and interactive services, remote monitoring and tele-medicine play a positive role in such a context and they can be utilized by hospitals as a tool to gain competitive advantage, to permit faster, and on-demand response to patient enquiries, to improve internal efficiency and productivity, and to reduce transaction cost.

Websites are one of the main channels of communication between hospitals and their stakeholders. Growing numbers of healthcare organizations are taking advantage of the communication and socialization opportunities offered by their websites, not only to present their services but also to promote loyalty. In Community Interaction criterion, we investigate suitable ways and instruments for interacting and fostering patients' participation and providing access to reliable and authoritative information. Hospital websites can promote the sharing of experiences of treatments and the adoption of healthy lifestyles.

Analyzing hospital websites becomes apparent that some technological aspects such as the simplicity or intuitiveness of navigating the website or the number of access channels are critical factors for patients when selecting e-health services. They help make a hospital website accessible and contactable for its patients. Technology criterion investigates aspects such as structural elements, accessibility compliance, navigability, ease of use, search engine, interactive responsiveness, reliability, security, response time, visual appeal, graphic style, appearance, etc.

**Figure 1:**
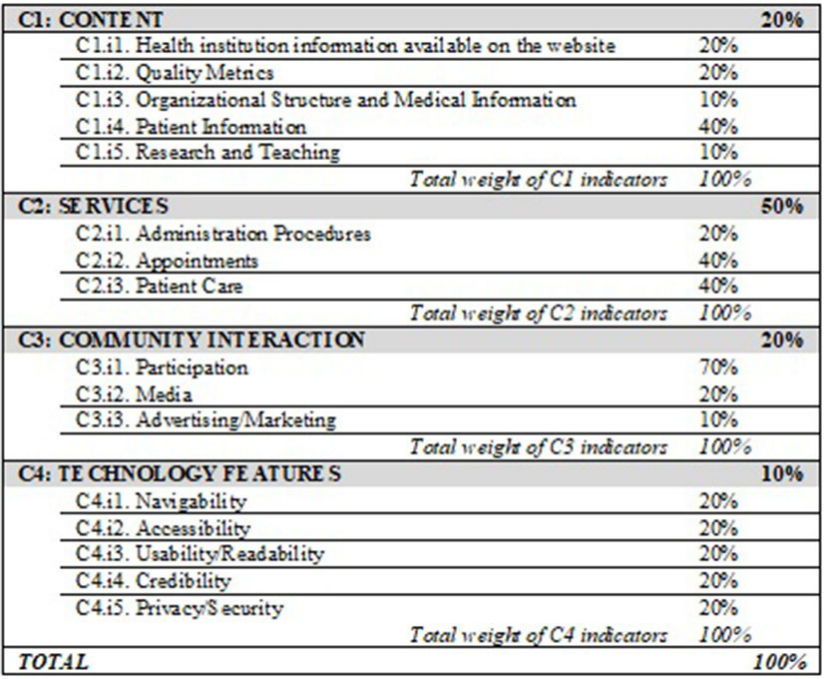
HSWAI Criteria, indicators, and relative weights.

Each HSWAI criterion includes a set of indicators, each of which has a specific weight (Figure 1) that illustrates its relative relevance to the overall assessment of the website (22). Each indicator is further subdivided into subindicators.

Subindicators of an indicator weight equally, being their weight obtained by formula (1).


(1) $$\begin{array}{l} \text{Subindicator weight = }(\text{1/}(number\_of\_applicable\_\\ \;sub\_indicators){)}^{*}\text{100}\% \end{array}$$

Taking into account these weights, an index that shows the level of maturity of the health institution website can be calculated. The calculation of the value of each indicator is obtained by formula (2).


(2) $$\frac{\sum values\;of\;all\;the\;sub\_indicators}{\text{n}umber\;of\;sub\_indicators\neq \;"not\;applicable"\;/total\;number\;of\;sub\_indicators}$$

The values of the four criteria are calculated by formulas (3), (4), (5) and (6).


(3) C1=20×  C1i1+20× C1i2+10× C1i3+40× C1i4+10× C1i5

(4) $$\begin{array}{l} C2=20\times \;C2i1+40\times \;C2i2+40\times \;C2i3 \end{array}$$

(5) $$\begin{array}{l} C3=70\times \;C3i1+20\times \;C3i2+10\times \;C3i3 \end{array}$$

(6) $$\begin{array}{l} C4=20\times \;C4i1+20\times \;C4i2+20\times \;C4i3+20\times \;C4i4+20\times \;C4i5 \end{array}$$

The final value of *i*_*HSWAI*_ is a value between 0 and 1 and it is obtained by formula (7).


(7) $$\begin{array}{l} i_{HSWAI}=20\times \;C1+50\times \;C2+20\times \;C3+10\times \;C4\; \end{array}$$

### The application procedure

The assessment takes place in 2019 through the direct observation of the health institutes' websites. For this reason, the first step in the application process is to identify the URL of the health institute main website. In addition, remarkably relevant is to identify the type of health institute that is being assessed. Some elements of HSWAI and some weights allocations vary slightly depending on the type of health institute: some sub indicators are not applicable for private health institutes; some indicators are just applicable to university teaching health institutes. For this reason, the type of health institute must be identified right at the beginning of the assessment process. Standardization of sub indicators weights' is achieved using formula (1).

For this study, 132 national Portuguese hospitals have been considered. Hospital selection is grounded on the list provided by the Portuguese Health Regulatory Entity on their website of National Health Assessment System (SINAS), which was the only official Portuguese hospital list at the time of the survey. The SINAS—National System of Evaluation of Health—is an assessment method of the overall quality system for health care providers establishments developed by the Regulatory Authority of Health in Portugal.

Data collection was based on direct assessment of the hospitals' websites conducted during July and August 2019 (23). The first stage in the data collection phase was to identify the hospital's website. After having the correct link, the assessment started through direct observation of the set of criteria, indicators, and sub indicators described. During the stage of data collection, value 1 was assigned to the presence of the considered sub indicator, value 0 to its absence, and NA if it was not applicable.

The study was performed by a group of two assessors, under the supervision of a third one (supervisor), who is an expert on the assessment process. This means that for each hospital website there are two values (one from each assessor) which were approved by the supervisor. In cases where the two assessors assigned different values to a specific sub indicator, this was signaled to them in order to be reassessed more thoroughly. In case the assessment discrepancy remained, the supervisor resolved which value was assigned to the subindicators.

Data was then treated to attribute one single value to each sub indicator, eliminating discrepancies and avoiding misclassifications. Assessors' commentaries were construed to facilitate this task and regarded as complementary information.

## Findings

**Figure 2:**
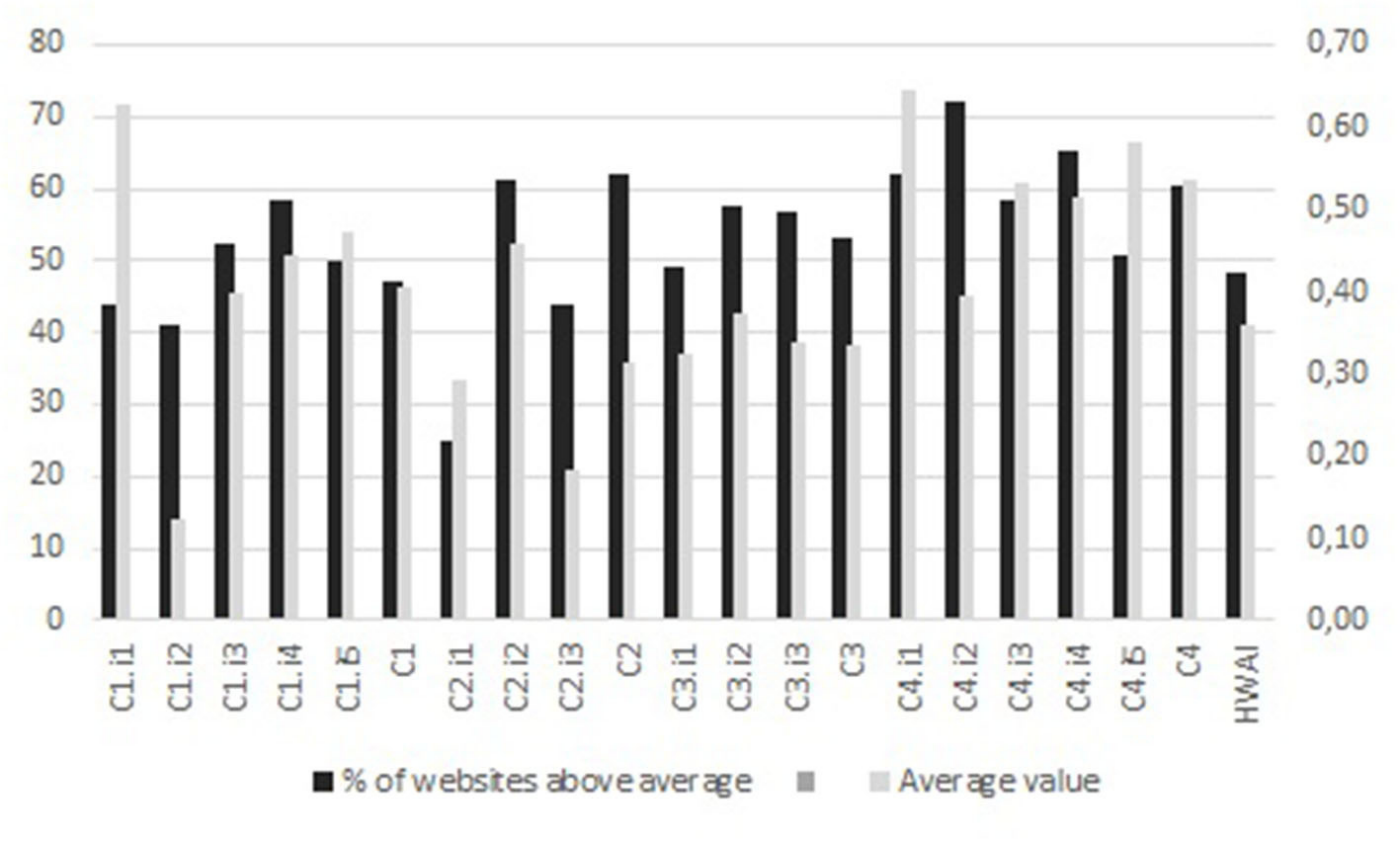
Criteria and indicators average values and percentage of websites that score above the average value.

This section presents the results of the application of the HSWAI to the Portuguese context for each of the identified criteria and indicators of the instrument. The average value is used in the study, to determine a single central value in a large set of discrete data of criteria, indicators and subindicators, for further analysis. Furthermore, the percentage of websites that score above the average value, in each criterion/indicator, is used to illustrate the hospitals websites performance in specific areas (Figure 2).

A detailed analysis for each criterion is provided in the next paragraphs.

### Content

The average value (AV) of Content criterion (C1) is 0.41 and 47% of Portuguese Hospitals (PH) score above the AV (Figure 1).

**Figure 3:**
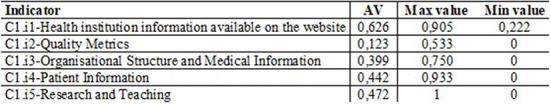
Average value (AV), maximum value (Max value), and minimum value (Min value) obtained by each indicator composing criterion Content (C1).

Concerning indicators' values (Figure 3), in Health institution information available on the website (C1.i1) indicator, 44% of PH score above the AV (0.63). Almost all websites present basic information related to hospital (e.g., management reports, name, logo, postal address, telephone/fax number, email, statement of purpose, complementary services, ways of reaching it, public procurement announcements, phone directory, institution history). Approximately half of them provide basic financial (VAT number), regulatory and certification data and few of them provide information regarding legislation issues, emergency situation information, open data and public procurement data.

Quality Metrics (C1.i2) is the indicator with the lowest performance in C1 criterion and has an AV of 0.12 and 41% of PH score above the AV. For this indicator, the only subindicators covered adequately are waiting time to be seen in the emergency room (79%) and number of institution beds disclosed (51%). The rest of the subindicators (e.g., waiting time consultation, waiting time surgery, results of patient surveys, number of internships, institution quality indicators) illustrate a low performance.

Organizational Structure and Medical Information (C1.i3) indicator has an AV of 0.4 and 52% of PH score above the AV. List of services available at the institution (95%) and departments or units providing user services (90%) are the only subindicators that are satisfied by almost all hospitals. Many websites provide a list of employed doctors (67%) and the possibility to read online or to download health-care booklets (64%). There is a lack of information in specific organizational structure features like organizational charts, personnel profiles and specific units' related information (e.g., services list, work hours, location).

Patient Information (C1.i4) has an AV of 0.44 and 58% of PH score above the AV. More than half cases provide patient privacy information, basic admission and insurance related information. The performance is worse in the area of specialized admission information.

Research and Teaching (C1.i5) has an AV of 0.47 and 50% of PH score above the AV. This indicator has the uniqueness that it was only assessed in hospitals that have a connection with universities and consequently include research and teaching capacity. Research related activities and library existence are fairly illustrated in most websites. Some shortcomings appear in specific library information (e.g., address, work hours, catalog).

### Services

In terms of Services (C2) criterion (Figure 2), the average value (AV) is 0.31 and 62% of Portuguese Hospitals (PH) score above the AV.

**Figure 4:**
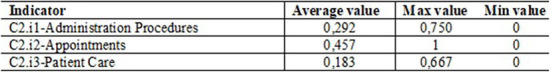
Average, maximum and minimum values obtained by each indicator composing criterion Services (C2).

Concerning indicators' values (Figure 4), Administration Procedures (C2.i1) has an AV of 0.29 and 25% of PH score above the AV. Hospital websites perform satisfactorily on provision of online forms (89%). They underperform in electronic payment (14%), possibility of forms downloading (13%) and on possibility of filled forms uploading (0%).

Appointments (C2.i2) have an AV of 0.46 and 61% of PH score above the AV. Although many of the websites provide the possibility to manage visits to outpatient consulting rooms (72%) and medical examination via web provision (61%), only few provide the possibility to manage admission via web (4%).

Patient Care (C2.i3) has an AV of 0.18 and 44% of PH score above the AV and it is the indicator with the lowest performance among all criteria. Several websites maintain an electronic directory with patient's records (41%), private area access with login and password authentication (37%), patient telemonitoring (30%) and private area access with citizen card or mobile digital key (18%). Few of the websites provide the possibility to request and obtain online medical prescription (17%), telemedicine services (15%), asynchronous communication with the doctor via e-mail (5%), synchronous communication with interactive communication tool (chat with a doctor) (1%) and none provides asynchronous communication with the doctor via a message exchange system.

### Community interaction

In terms of Community Interaction (C3) criterion (Figure 1), the average value (AV) is 0.34 and 53% of Portuguese Hospitals (PH) score above the average value (AV).

**Figure 5:**
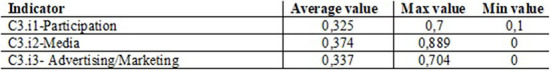
Average, maximum, and minimum values obtained by each indicator composing criterion Community Interaction (C3).

Participation (C3.i1) indicator (Figure 5) has an AV of 0.33 and 49% of PH score above the AV. Although most of the websites provide the possibility of information requests via the web (99%), suggestions via web (75%) and complaints via web (71%), only few of them provide FAQ (39%), voluntary associations that work at the institution (27%) and fewer provide opinion polls (5%), associations for the defense of patients' rights (4%), communication with the institution via chat (2%) and none of them provides a discussion forum.

Media (C3.i2) has an AV of 0.37 and 58% of PH score above the AV. 78% of the websites provide an up-to-date news/events schedule/newsletter, 74% of them provide institution news, 72% of them provide links to other websites of interest and 70% have a media presence. Websites perform poorly in the area of public relations where only 18% provide the related email address, 17% provide a telephone or fax number, 5% the location, 2% the working hours and none provides a possibility of virtual visit to the institution.

Advertising/Marketing (C3.i3) has an AV of 0.34 and 57% of PH score above the AV. Information about job opportunities at the hospital is provided in 70% of the websites. Sixty four percentage of them provide a Facebook link, 45% of them a YouTube link, 27% a LinkedIn link, 6% a Twitter link and 20% of them some other social network link. Inadequate performance is observed in the following areas: information on how to make a donation to the hospital (1%), disclosure of website sponsors and investors (0%), differentiation between advertising and contents (0%) and advertising is not contradictory with respect to the website contents (0%).

### Technology features

Technology Features (C4) criterion is the best performing criterion, the average value (AV) is 0.53 and 61% of Portuguese Hospitals (PH) score above the AV (Figure 1).

**Figure 6:**
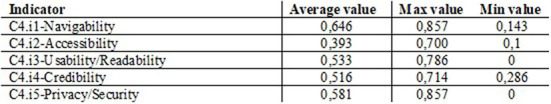
Average, maximum and minimum values obtained by each indicator composing criterion Technology Features (C4).

Navigability (C4.i1) indicator has an AV of 0.65 and 62% of PH score above the AV (Figure 6). The majority of the considered websites provide functioning interwebsite and intrawebsite links, the active part of the site appears on browser title bar, the website name appears on browser title bar and interwebsite links show a full description of the linked website. On the contrary, they do not perform satisfactorily in the following areas, interwebsite links are distinguished from intrawebsite links and best browser version for the website is indicated.

Accessibility (C4.i2) has an AV of 0.39 and 72% of PH score above the AV. Almost all websites are listed on the first page of results after a Google search, they are compatible with the 3 most used mobile browsers and the 3 most used browsers in the country. Almost half of them provide specific and meaningful description via the META/description tag for individual subpages. The downfall is that most of the websites do not provide accessibility (WCAG) and mark-up validation (W3C) symbols on the main page.

Usability/Readability (C4.i3) has an AV of 0.53 and 58% of PH score above the AV. Almost all websites include individual subpages with specific and meaningful titles, provide a menu structure for navigating the subpages, graphics open conveniently, illustrations/pictures/photos accompany text to assist description, and provide technological sophistication with the use of APIs or widgets. In most of them, the website layout is responsive and provide a search engine. Almost half of them include a website map. A small percentage loads relatively quickly, the pages can be printed and include content in foreign languages. Very few websites offer means to adjust the contrast of textual information for visitors with visual impairments, include pop-up advertising and offer means to adjust the text size without compromising the functionality of the website.

Credibility (C4.i4) indicator has an AV of 0.52 and 65% of PH score above the AV. Almost all websites are grammatically correct and do not have spelling errors. Most of them provide content update indications and specific date of last website update. On the contrary, they lack interest conflict declaration or declaration of non-conflict, certification indication and webmaster characteristics.

Privacy/Security (C4.i5) indicator has an AV of 0.58 and 51% of PH score above the AV. The majority of the websites provide copyright notice, a website privacy policy, include a cookie policy and indicate the ownership of the site. Approximately half of them provide general disclaimers and website encryption. The responsible of the website content cannot be identified in the majority of them.

## Discussion

The growing demand of patients to access health information and receive services online (24), has led hospitals in Portugal to create and maintain well-designed websites that adhere to international technological standards. In the remainder of this section, the survey's findings are discussed and recommendations relevant to assessment researchers, policy makers, hospital managers and website designers are provided.

To the best of our knowledge, no prior study evaluated Portuguese hospital websites thoroughly by examining the content, services, community interaction and technology features criteria. Although only a part of all hospitals in Portugal was assessed, this can be considered as representative. Our findings highlight that the evaluated websites focus primarily on promoting content and satisfying technology features rather than providing electronic services or engaging and communicating with patients.

Portuguese hospitals' websites provide smooth and adequate design and functionality, covering most of the emerging technological requirements regarding accessibility, usability, readability, credibility, privacy and security aspects. Conformance with accessibility guidelines, proper visual adjustments, conflict declaration, certification indication and webmaster characteristics are the only features where they underperform.

The results also clearly indicate that the hospitals are mostly using their websites as information-giving, since they provide static information such as hospital location and reachability, organizational structure, information regarding services and doctors, research activities, etc. Although most of the information provision needs are covered, some gaps appear in specific areas (e.g., quality metrics, specific hospital procedures).

Excluding provision of online forms and visits to outpatient consulting rooms' management, electronic services are not covered properly. Underperformance appears in electronic payment and automated administrative transactions. By improving their performance in procedures and patient care indicators, hospital websites may help substitute traditional administrative and clinical processes with web-based interactive processes that will be more responsive and transparent to the users. Hardly any hospital website allows interaction between doctors and patients. This is a crucial component of patient service and health information sharing. The websites can be geared up not only to allow doctors and patients interaction, but also to encourage patient-to-patient and doctor-to-doctor interaction. In addition, with emerging information and technology development, and the evolution of new medicines and medications, it becomes critical that even other partners of health service chain, such as pharmaceutical or health equipment companies discuss and share information with healthcare practitioners and patients in social networking platforms. This transparency enhances trust between patients, doctors, pharmaceutical and insurance companies. Furthermore, awareness will benefit people in general in terms of availability of various treatments, which can help them make more targeted and updated decisions. This will principally lead to mutual trust and responsibility sharing among patients and doctors.

Traditional participation mechanisms (e.g., information requests and complaints submission), media channels (e.g., news, events, newsletters) and job opportunities are generally sufficiently provided. However, not much attention has been given to use health sector websites more interactively and use them as communication channels and knowledge sharing tools. To achieve this, hospitals must provide both a platform for potential customers that are patients seeking guidance, and also engage them in the exchange of information as well. This aspect is absent on most of the hospital websites.

Furthermore, if Portuguese hospitals do not take the advantage of web technologies to share accurate information and do not facilitate exchange between patients and physicians, or even amongst patients themselves, then hospitals' role in many future healthcare decisions might be greatly reduced. By designing interactive websites, healthcare institutions will move toward a healthy and productive competition. This will also be beneficial in terms of restraining the escalating healthcare costs. Patients can access and consider different treatment methods and their costs and thus make more informed decisions. Furthermore, by upgrading their performance in participation and advertising/marketing indicators, hospitals can gain feedback, which in turn will improve health services.

## Conclusion

The public value perspective of online health services has gained significant momentum over the past decade. However, the evaluation from a user point of view is still very limited. In this article, a comprehensive framework for hospital websites assessment has been applied to the Portuguese context. The hospital websites have been assessed using HSWAI suggested metrics.

Portuguese hospitals (from ERS's list) websites were assessed, and recommendations offered for healthcare organizations and website developers to improve their individual design, information dissemination, services and operation. Hospital managers can incorporate this website assessment to determine which aspects and features are more essential for their institution. Several countries worldwide have similar healthcare systems to Portugal, face similar challenges, and share similar healthcare issues. As such, any acquired information about the status of hospital websites in Portugal will in turn benefit them.

The study has theoretical, practical, and policy implications contributions. From a theoretical perspective, the study makes a knowledge contribution to the limited literature on hospital websites assessment by suggesting an assessment instrument and by providing empirical evidence from its application. From a practical viewpoint, the study identified specific areas of concern in terms of delivering value via hospital websites. Lastly, the study provides a policymaking support tool for health sector decision makers.

This is the first study to explore the websites of general hospitals in Portugal. These websites are a potentially useful source for the citizenry. However, the study indicates that the existing websites of Portuguese hospitals have several limitations. The majority of the hospitals performed well in the website technological features and content criteria. With respect to electronic services or engaging and communicating with patients' criteria, only a limited number of hospitals demonstrated a satisfactory performance.

The areas that these websites need to enhance and improve include: quality metrics, specific hospital procedures, electronic payment and automated administrative transactions, allowing interaction between doctors and patients, interactive participation mechanisms, search tool, online appointments, web based feedback, electronic enrolment process, language, privacy & security rules, etc. In order to improve the abovementioned aspects, a definite position must be adopted by the hospitals' websites. The purpose of hospital websites is to serve the public and each patient. So great efforts shall be made for service and management. Experiences and achievements from successful cases worldwide shall be acquired and used for reference. Efficient and user friendly processes for the public and the patients specifically, shall be developed under the existing technical infrastructure. Administration and maintenance of hospital websites shall be enhanced, placing the appropriate weight to each one.

This study reveals variability with respect to the Portuguese hospitals. Health sector websites could be significant sources of accurate information and patient guidance when they meet the above standards. As people increasingly use digital means for detailed health information, general hospitals need to meet the increasing standards and demands of healthcare consumers. This achievement requires the concerted efforts of the managerial staff of each hospital, the medical professionals, the website developers, and the website administrators.

Besides the identified results, there are several limitations to this study. This was an assessment applied to Portuguese conditions. In the next step, the assessment instrument will be applied in other countries worldwide and transnational analysis will take place leading to more interesting and valuable information.

This field has high potentiality for future investigations and research directions. More surveys on patients' demands, technological aspects, hospitals' operational and management needs and their relative indicators are planned for the future, especially involving various stakeholders in the research.

## Data availability statement

The original contributions presented in the study are included in the article/supplementary material, further inquiries can be directed to the corresponding author.

## Author contributions

All authors listed have made a substantial, direct, and intellectual contribution to the work, and approved it for publication.

## Conflict of interest

The authors declare that the research was conducted in the absence of any commercial or financial relationships that could be construed as a potential conflict of interest.

## Publisher's note

All claims expressed in this article are solely those of the authors and do not necessarily represent those of their affiliated organizations, or those of the publisher, the editors and the reviewers. Any product that may be evaluated in this article, or claim that may be made by its manufacturer, is not guaranteed or endorsed by the publisher.
